# A Novel Data Association Algorithm for Tracking and Localization Using Segmented Data Analysis

**DOI:** 10.3390/s26196161

**Published:** 2026-09-29

**Authors:** Maria Volkova, Alexey Romanov

**Affiliations:** Institute of Artificial Intelligence, MIREA—Russian Technological University, Moscow 119454, Russia; volkova_m@mirea.ru

**Keywords:** multi-object tracking, data association, updraft localization, Kalman filtering, parallel algorithms, sensor data processing

## Abstract

Multiple object tracking is used in a range of applications, including autonomous control of various vehicles and sports analytics. The main challenges in developing tracking systems are detection errors and computational load that scales with the number of tracked objects. Therefore, the problem remains highly relevant. This paper formalizes the generalized multisensor tracking task for multiple objects. To address this task, a novel algorithm for object tracking and localization is proposed. It combines cluster analysis, relevance estimation for object data, and a set of Kalman filters that can be computed independently and in parallel. The algorithm operates at the data association stage and uses segmented data on object positions and additional features. The architectural design ensures compatibility with both modern methods and traditional approaches for detection and segmentation. The approach is suitable for a wide range of applications. The algorithm was tested on the publicly available APIDIS dataset, allowing direct comparison with existing methods and demonstrating competitive performance with a MOTA score of 81.8%. The feasibility of the algorithm for thermal updraft localization was demonstrated by tracking soaring objects using a custom dataset of videos recorded by a paragliding pilot. The results confirm the potential of this approach for deployment on autonomous unmanned gliders.

## 1. Introduction

Tracking systems are widely used for various applications, including the implementation of augmented reality technologies in match broadcasting systems, automated performance evaluation in sports, the development of autonomous control of unmanned vehicles, traffic management, and others. At the same time, regardless of the sensor system or types of observed objects, operating conditions and common input data suggest the possibility of using unified solutions.

The multiple object tracking problem is generally solved in two stages [1]: the first stage involves preprocessing sensor data, including segmentation into different objects and determining their features, such as appearance (including pattern recognition), a process known as detection. The second stage is data association, which consists of matching the obtained data with previously known objects to estimate and analyze their trajectories to solve the final applied task. However, in most studies, the main focus is often on the segmentation stage for the following reasons:

1. The quality of the segmentation result directly affects the possibility of further data matching.

2. The tracking stage can be reduced to a trivial approximation of the recognized object trajectories under conditions of high-quality segmentation and pattern recognition.

3. In recent years, the development of neural network approaches and machine learning methods has provided many new tools to solve segmentation and recognition problems [2], which have generated considerable interest in the research community.

In practice, it is often impossible to determine the features of all objects due to various factors, such as difficult observation conditions or high object movement speeds. Therefore, it is important to develop a data association algorithm for tracking and localization that can adapt to incomplete or inaccurate data.

At the same time, various specialized solutions are being actively developed, some of which are designed to work only with a certain type of sensor [3]. Some studies combine detection and tracking, making them codependent [4]. Some solutions are based on assumptions (for example, the location of all moving objects in a single plane) [5]. Such studies typically focus on solving specific applied tasks and do not aim at universality, which is in demand, for example, when changing the type of observed objects. At the same time, regardless of the sensor type used or the assumptions made, the whole variety of private solutions is aimed at solving the main challenges at the tracking stage:

1. Data matching in the presence of incomplete or erroneous input data.

2. The increasing complexity of computational calculations with an increase in the number of observed moving objects.

The first challenge is the errors resulting from segmentation due to the following reasons: environmental conditions (insufficient lighting or low contrast), low sensor resolution, and imperfections in the chosen detection algorithm. These include occlusion and false positives or false negatives in object segmentation. The second one is primarily due to the combinatorial nature of the data association problem, which leads to exponential growth in the required computational resources. For instance, the Multiple Hypothesis Tracking (MHT) algorithm critically requires pruning mechanisms to limit the number of hypotheses.

This research aims to develop a unified multiple object tracking algorithm suitable for diverse applications. The algorithm is designed to track moving objects effectively and is invariant to the number of sensors employed. It is completely independent of the segmentation method and does not rely on the number or specific type of additional object features. The paper evaluates the tracking algorithm using two distinct datasets acquired from a stationary multi-camera system and a single mobile camera. The proposed approach delivers quality characteristics comparable to existing solutions.

The main contributions of this paper are as follows:

- A generalized tracking task statement applicable to various problems without specifying the number and types of sensors or detected objects.
- A novel data association algorithm for tracking and localization that adapts to a dynamic number of targets and is applicable to diverse tasks. The algorithm is robust to segmentation quality, achieving a tracking performance of over 81% MOTA even when employing a legacy segmentation technique.
- Experimental validation of thermal updraft localization, which reflects the real-world tactics of professional paragliders who prioritize the actual concentration of soaring objects over statistical data available through specialized services.

The rest of the paper is organized as follows. Section 2 briefly reviews related works. A multisensor tracking task statement is described in Section 3. Section 4 presents a novel algorithm for tracking multiple objects using segmented data analysis. The experimental results are given in Section 5. Section 6 discusses the main results of the research and formulates the conclusions.

## 2. Related Works

Today, numerous methods and algorithms exist for multiple object tracking based on segmented data (also called observations) from a sensor system. Prominent examples of classical approaches include the Joint Probabilistic Data Association (JPDA), MHT algorithm, the Probability Hypothesis Density (PHD) filter, the Multi-Bernoulli filter, and the Labeled Multi-Bernoulli (LMB) filter [6]. The JPDA offers low computational complexity. However, this approach requires a fixed and a priori known number of targets. MHT exhibits high robustness against false positive detections. This method evaluates possible measurement-to-track association hypotheses. Consequently, this evaluation leads to significantly higher computational costs. Both PHD and Multi-Bernoulli filters successfully estimate the target count and their current states. Nevertheless, these foundational algorithms do not inherently connect object coordinates over consecutive time steps. External tracking algorithms are required to form continuous trajectories. As an advancement, the LMB modification introduces unique labels. Additionally, this method ensures high tracking accuracy. However, study [7] demonstrates that the LMB filter is unsuitable for tracking dense target clusters. Thus, these classical algorithms served as the baseline for the development of modern advanced multi-object tracking methods.

Modern methods can be categorized in various ways, which are presented in reviews [1,8] and others. The study [8] focuses on the data association stage and covers the legacy and new tracking techniques. The authors propose classifying the literature into four groups according to the structure and mechanism of the association. Of particular interest are the methods of the first and second groups, namely probabilistic and hierarchical methods, which exhibit superior tracking quality and performance metrics, and are thus more widely represented in the current literature.

Probabilistic (bayesian) methods provide a mathematical framework for sequentially updating object state estimates as new measurements arrive. Based on this framework, specialized algorithms account for false alarms, missed detections, data association ambiguity, and time-varying numbers of targets. Practical implementations of these approaches utilize the Kalman filter and particle filter combined with JPDA and MHT data association algorithms.

The Multimedia and Vision group has developed a tracking method using a particle filter [9,10]. This approach does not require prior knowledge of the number of objects being tracked. Instead, it employs the Mean-Shift clustering method, which operates without needing information about the number of clusters beforehand. In [11], an approach to tracking objects that are similar in appearance is proposed based on the maximum a posteriori expectation-maximization algorithm (MAP-EM), which is based on the EM algorithm, in which the maximum likelihood of the parameters of a probabilistic model is estimated in terms of hidden variables. This approach provides a high MOTA score for this group of methods—85.5%. In [12], the authors propose an original approach to combat distortions in the predicted trajectories of observed objects as a result of occlusions, based on the combination of a Kalman filter and a particle filter. The authors note that this approach increases localization accuracy. However, its use is limited by several conditions: objects are well segmented, move at low speeds, and the number of particles is correctly selected. The paper also compares the performance of the Kalman filter and the particle filter and concludes that the Kalman filter processes data 1.13 times faster. In the study [13], a solution to the occlusion problem is proposed. The formulation of the MAP problem for the data association deserves special attention.

Hierarchical methods have a multilevel structure of interaction of various tracking algorithms to match observation–object or observation–tracklet pairs (a sequence of already compared observations for a specific object obtained over a certain period of time). The number of levels may vary [14,15]. Neural networks (R-CNN, LSTM, etc.) are often used in this line of work. The main focus is on appearance-based processing and the calculation of similarity metrics, while motion parameters are also taken into account and analyzed using the Kalman filter [14]. Unlike probabilistic methods, this approach is applied without a PDA. Many studies use high and low confidence scores for comparison [15], which are formed as a result of the first iteration of calculations (at the first level), and observations for pairs with a low score are the initial data for repeated calculations with new conditions or for other algorithms (at subsequent levels). In [14], the four-step method is presented, where two types of matching are used using the Hungarian algorithm: observation–tracklet and tracklet–tracklet, including global and local matching. The approach provides high tracking accuracy, but processing one frame takes 60 ms.

The works mentioned above have become the starting point for many researchers, and the algorithms proposed in them for tracking moving objects have received many modifications, along with the development of hybrid approaches. In [5], a hybrid method is presented that combines constructing an occupancy map to locate players on the field with extracting key features (such as jersey colors and player numbers) for object classification. This information is subsequently integrated into a tracking method based on the K-shortest path algorithm. The solution to the matching problem in it is inextricably linked to segmentation. The occupancy map is formed directly at the stage of player image recognition, significantly limiting the use of this algorithm and its derivatives in tasks where measuring devices other than video cameras are used as sensors. A MOTA score of 100% deserves a separate discussion. This high indicator was achieved because all signs for all observed objects were correctly identified throughout the sequence of frames. In practice, such frame sequences are extremely rare, as they require shooting conditions close to ideal. The LSTM-based method [16] processes data at 30 frames per second (fps) but achieves a MOTA score of 75.2% on the same dataset used in [5]. In [17], an approach was proposed that takes into account an occupancy map and visual information (including the poses of the players), which ensures a MOTA score of 81.1%. However, the authors note the high computational complexity of the proposed approach compared to existing works. Based on the analysis, the following conclusion can be drawn: the structure of hierarchical methods allows for high tracking accuracy, but they are inferior to probabilistic methods in terms of performance due to an increase in the number of levels of interaction between algorithms. Thus, a promising development is integrating a probabilistic approach with the Kalman filter into a hierarchical structure to account not only for motion parameters but also for object features. A tracking method based on the analysis of object parameters is also preferable due to the great prospects for increasing localization accuracy.

## 3. A Multisensor Tracking Task Statement

Table 1 summarizes the notations used throughout this paper along with their descriptions.

A tracking system includes *K* sensors, where K≥2. The sensor calibration parameters are assumed to be known, regardless of whether the sensors are stationary or their displacements are known. The system observes a scene containing *N* moving objects, where N>1. Each moving object is presented by a state vector $x_{i}={\left(S,V,a\right)}^{T}$. The sub-vectors *S*, *V*, and *a* denote the spatial position (coordinates of the object centroid), velocity, and acceleration components, respectively, along each coordinate axis. Furthermore, each moving object is characterized by an additional feature vector $F_{i}$, which comprises a set of numerical attributes such as color, shape, size, and temperature. The number of features is predetermined, taking into account the types of sensors employed and the corresponding segmentation algorithms.

The source data for tracking are the results of the segmentation algorithm for each *k*-th sensor and are represented as two vectors:

(1) ${\hat{S}}_{k}=\left({\hat{S}}_{\mathrm{kj}}:j=1,2,\ldots ,J_{k}\right)$, where ${\hat{S}}_{\mathrm{kj}}$ denotes the centroid coordinates of the *j*-th detected object from the *k*-th sensor, segmented based on its measurements,

(2) ${\hat{F}}_{k}=\left({\hat{F}}_{\mathrm{kj}}:j=1,2,\ldots ,J_{k}\right)$, where ${\hat{F}}_{\mathrm{kj}}$ denotes the numerical attribute of the additional feature for the *j*-th observation from the *k*-th sensor.

The multisensor tracking task is formalized as follows. A system of mutually synchronized sensors used to monitor moving objects is considered. Each object is represented by a state vector $x_{i}$ and a dynamic model $x_{i}^{\prime }=Ax_{i}$, where i∈1,…,N. *N* denotes the time-varying number of objects, *A* is the state transition matrix, and $x_{i}^{\prime }$ is the predicted state vector. For each sensor *k*, the measurements are automatically segmented. At each discrete time step with a given sampling period, a set ${\hat{O}}_{k}$ is formed, comprising $J_{k}$ observations. Each observation in ${\hat{O}}_{k}$ includes a vector of additional features ${\hat{F}}_{k}$ and a bounding box centroid coordinate vector ${\hat{S}}_{k}$, which was corrupted by uniformly distributed random measurement noise. Consequently, the task of tracking moving objects is: (i) to determine the number of observed objects *N* at each discrete time step; (ii) to match each observation with its corresponding object index; and (iii) to obtain for each *i*-th object an estimate of its state vector $x_{i}$ that minimizes the standard deviation between the obtained state estimates and the matched measurements.

The number of observations $J_{k}$ may differ across synchronously obtained measurements from different sensors as well as across sequential measurements from a single sensor. This variation occurs because the number of segmented objects changes between frames. For example, if two objects are occluded, they may be segmented as a single object. An object may also leave the sensor’s field of view or, conversely, enter it. Moreover, observations obtained for the *i*-th object by the same sensor during different measurements may actually correspond to different objects. The segmentation procedure does not guarantee preservation of either the number or the order of the resulting segments. The obtained observations cannot be used directly to solve the tracking problem.

In connection with the above, it is proposed to divide the concepts of segmented images of objects into two types: true and observed. **The observed image** of the object is a set of actual measurements ${\hat{O}}_{k}\left(x_{j}\right)$ obtained as a result of the segmentation procedure according to the *k*-th sensor data, where k=1,…,K. ${\hat{O}}_{k}\left(x_{j}\right)$ probably corresponding to the object $x_{i}$. **The true image** of the object is a set of data corresponding to the object located in the sensor’s work area $O_{k}\left(x_{j}\right)$. The true image of an object takes into account only the fundamental properties of the sensor. It does not depend on the imperfections of its specific implementation or noise and distortion associated with the current environmental conditions. With the correct definition of the true image in the coordinate system of each sensor, it is possible to estimate the spatial position of the object in the global coordinate system. An observed image from segmentation may represent at least one true image of an object. When an occlusion occurs, one observed image can correspond to several true images. If segmentation failed, some true images might not appear as observed images at all. False positives introduce observed images without matching true image. Thus, ${\hat{O}}_{k}\left(x_{N}\right)=\left\{{\hat{S}}_{\mathrm{kj}},{\hat{F}}_{\mathrm{kj}}\right\}$ is the *j*-th observation obtained by the *k*-th sensor, which is hypothesized to correspond to the object $x_{N}$.

**Figure 1:**
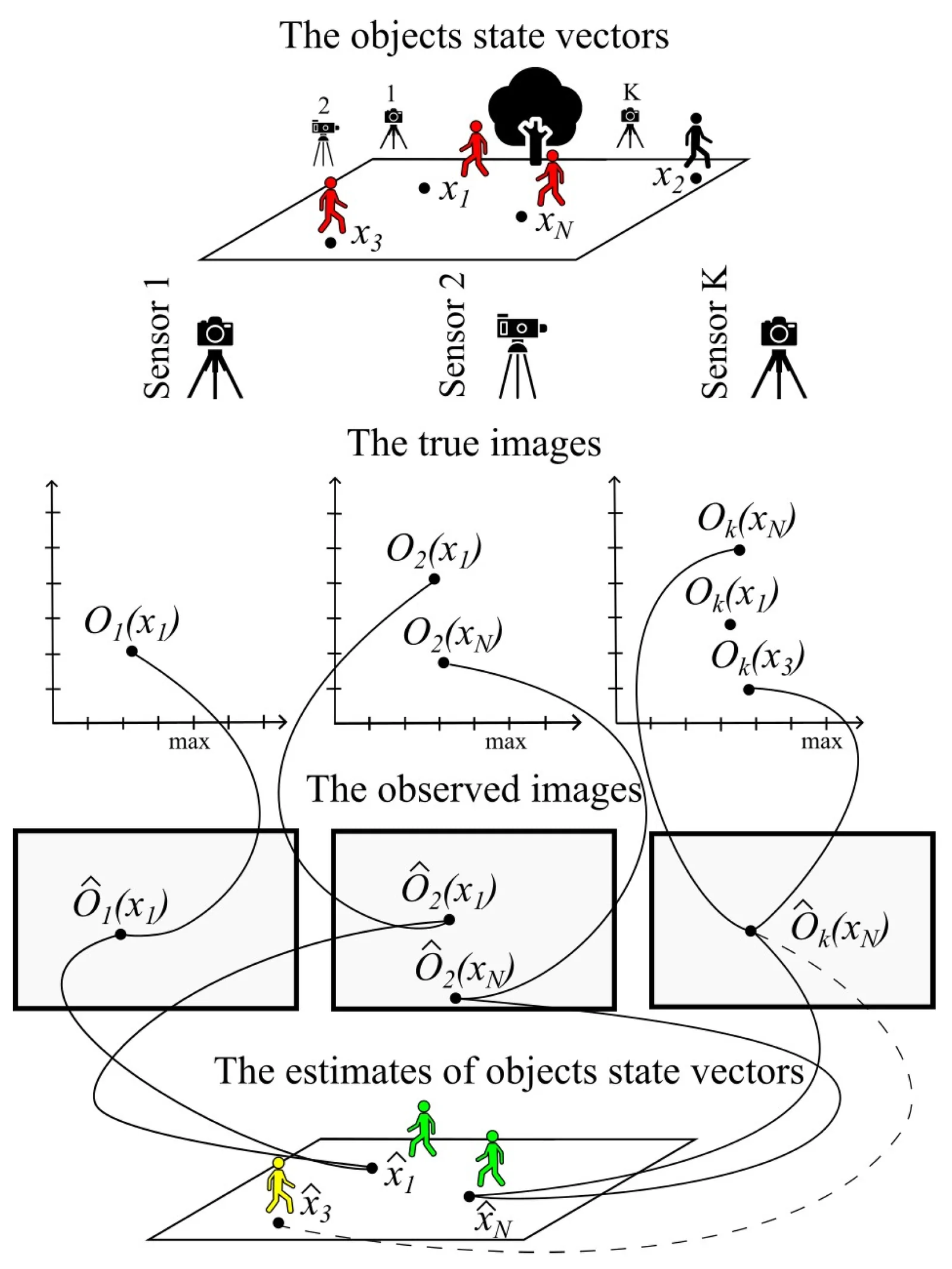
Correspondences between true and observed images of objects in solving the tracking task.

Correspondences between the true and the observed object images in solving the tracking task are shown in Figure 1. In this example, the objects $x_{1}$, $x_{3}$ and $x_{N}$ are within the field of view of three sensors. The object $x_{N}$ is located in the working zone of sensors 2 and *K*: $O_{2}\left(x_{N}\right)$ and $O_{k}\left(x_{N}\right)$. The observed images ${\hat{O}}_{2}\left(x_{N}\right)$ and ${\hat{O}}_{k}\left(x_{N}\right)$ have been successfully found for it, from which its three-dimensional coordinates have been reconstructed.

Three objects are located within the field of view of sensor *K*. The object $x_{1}$ is obscured by a tree, so no observed image is obtained for it. Objects $x_{3}$ and $x_{N}$ are close to each other in the image plane. Consequently, they are segmented as a single observed image, which is designated as ${\hat{O}}_{k}\left(x_{N}\right)$. This occurs because the object $x_{N}$ is located closer to the camera and occupies a significantly larger bounding box area. Thus, the coordinates of object $x_{3}$ cannot be reconstructed at the current time step.

To address the challenge of tracking moving objects, it is essential to compare the observed images of these objects with predictions of their true image at each iteration of sensory data processing. Additionally, it is crucial to identify the moment when new objects enter the sensor system’s field of view. Based on the results of this comparison, the state vector for each object can be estimated. This paper presents a novel algorithm for multiple object tracking that uses segmented data analysis to enhance accuracy and efficiency.

## 4. A Novel Data Association Algorithm for Tracking and Localization

A novel algorithm for multiple object tracking in the global coordinate system aims to match the true images with the observed images of the object $x_{i}$. Pre-calibration of all sensors before starting work is a prerequisite for the operation of the algorithm. It involves determining the relative linear and angular displacements of the sensor coordinate systems and selecting the origin of the global coordinate system. It is assumed that the position of the sensors relative to each other does not change during the entire operation time, or that their movements are known.

The focus is on processing motion parameters rather than on appearance models and calculating similarity metrics for various features, although these factors are considered in the final assessment of correspondences. This approach allows maximum abstraction from methods for segmenting moving objects, resulting in the exclusion of the segmentation stage from the algorithm’s structure.

**Figure 2:**
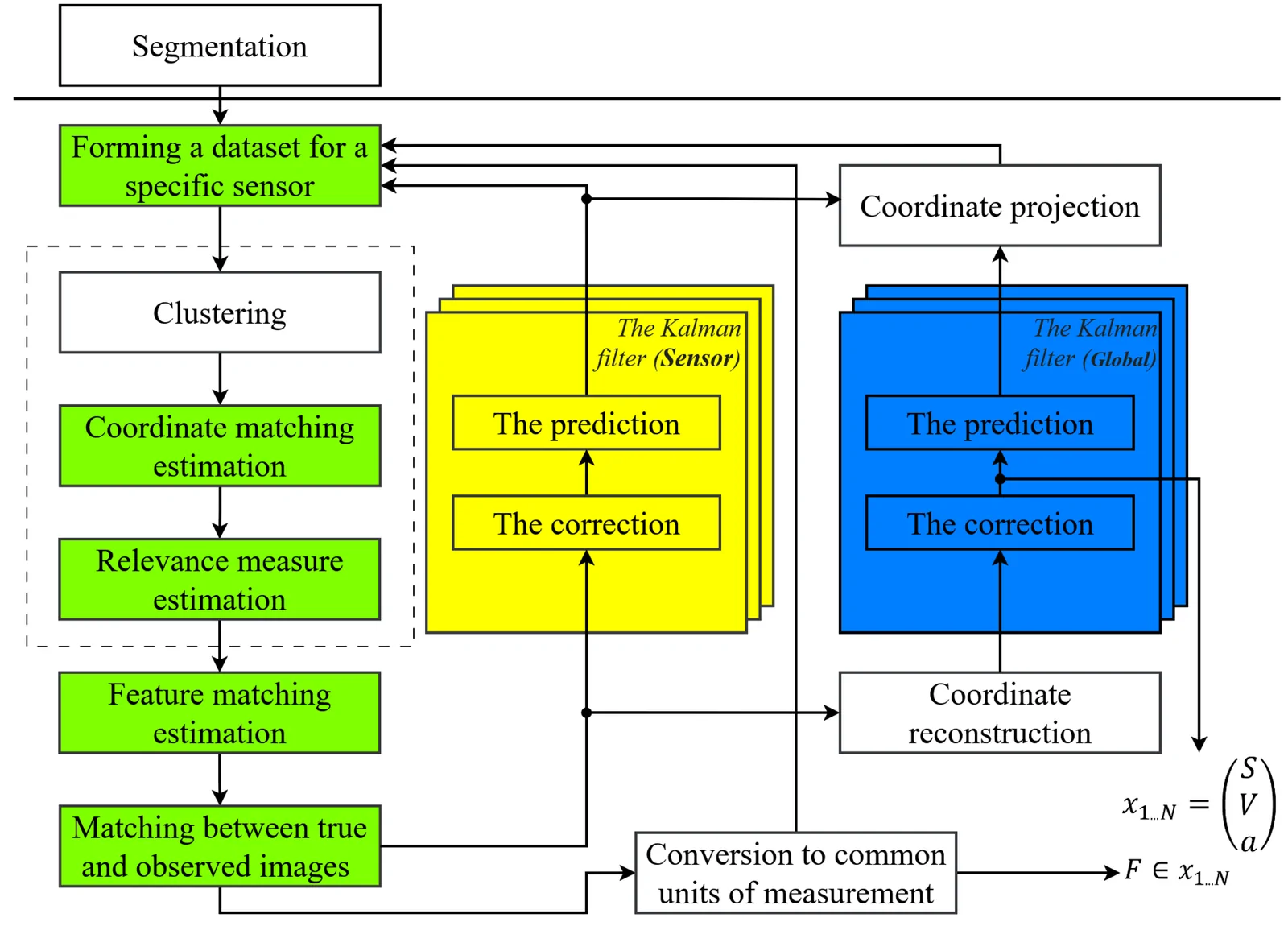
The structure of the algorithm for multiple object using segmented data analysis.

The structure of the proposed algorithm is shown in Figure 2. Novel blocks are marked in green and described in detail below. The dashed line groups the algorithm steps (clustering, coordinate matching estimation and relevance measure estimation), as all operations are performed on object coordinates.

To determine the relative positions of the true images of objects and to predict their future locations, two sets of mutually independent Kalman filters are employed. These filters estimate coordinates in both the global coordinate system and the sensor coordinate system. The Kalman filters for predicting the object coordinates in the global coordinate system are shown in blue, and those in the sensor coordinate system are highlighted in yellow. A separate Kalman filter is used for each object, with the equations provided in [18].

The output of this tracking algorithm is an object model that consists of a single identifier, which combines a state vector obtained from the Kalman filter correction unit for the global coordinate system with a set of additional features derived directly from sensory data, standardized to common units of measurement. The first element in the state vector $x_{1\ldots N}\left(S\right)$ is used directly to track moving objects within the global coordinate system.

The steps of the algorithm after receiving the segmented data are as follows.

**1. Forming a dataset for a specific sensor:** At this step, a set of object coordinates and features is formed for matching within the corresponding sensor. Coordinate matching is performed first. The dataset used is ${\hat{C}}_{k}=\left\{\bar{S},S^{\prime },{\hat{S}}_{k}\right\}$, where

- $\bar{S}$ is the set of predicted coordinates in the sensor’s coordinate system;
- $S^{\prime }$ is the set of projected predicted coordinates;
- ${\hat{S}}_{k}$ is the set of coordinates obtained by the *k*-th sensor at the measurement instant *t*.

Object indices are known for sets $\bar{S}$ and $S^{\prime }$, having been determined at the measurement instant t−1. The cardinalities of the sets are $|\bar{S}|=N$, $|{\hat{S}}_{k}|=J_{k}$, and $|S^{\prime }|=N\left(l+1\right)$, where l∈0,1,…,K−1 is the number of sensors from which the predicted coordinates are projected; the term “+1” accounts for the projection from the global coordinate system. The number of elements in each set may change at each iteration. Three distinct cases are possible, which are addressed in the subsequent stages:

1. All objects within the sensor’s field of view have been successfully segmented: $N=J_{k}$.

2. Erroneous segments are present or a new object has entered the sensor’s field of view: $N<J_{k}$.

3. Not all objects have been segmented, or an object has left the sensor’s field of view: $N>J_{k}$.

These conditions can be combined.

**2. Clustering:** Cluster analysis of the dataset ${\hat{C}}_{k}$ can be performed using the Mean Shift method [19] or other clustering methods that do not require the number of clusters to be specified in advance. The result is a set of clusters $C=\{C_{n}:n=1\ldots U\}$, where *U* is the total number of clusters. The set $C_{n}$ includes observations from ${\hat{S}}_{k}$ and $c_{i}$, where $c_{i}$ comprises the predicted coordinates from $\bar{S}$ and the projected predicted coordinates from $S^{\prime }$, all sharing the same object index *i*.

**3. Coordinate matching estimation:** The matching score $p(O_{k}\left(x_{i}\right);{\hat{S}}_{k_{j}})$ quantifies the correspondence between the *j*-th observation ${\hat{S}}_{k_{j}}$ of the *k*-th sensor and the true image of object $x_{i}$. It is calculated according to (1).

(1) $$p\left(O_{k}\left(x_{i}\right);{\hat{S}}_{k_{j}}\right)=\left\{\begin{array}{cc} 1, & \mathrm{if}\;|c_{i}|\;=l+2,\\ \frac{|c_{i}|}{l+2}, & \mathrm{if}\;0<\;|c_{i}|\;<l+2,\\ 0, & \mathrm{if}\;|c_{i}|\;=0. \end{array}\right.$$

Depending on the number of observations in the cluster $C_{n}$, one of the conditions in Algorithm 1 will be applied.
**Algorithm 1** The pseudocode of the coordinate matching estimation**Input:** $C=\{C_{n}:n=1\ldots U\}$, *l* **Output:** $p(O_{k}\left(x_{i}\right);{\hat{S}}_{k_{j}})$$p(O_{k}\left(x_{i}\right);{\hat{S}}_{k_{j}})=0$**for **n=1 to *U* **do**     **if** ${\hat{S}}_{k_{j}}\in C_{n}$ **then**         **if** $|c_{i}|=l+2$ **then**             $p(O_{k}\left(x_{i}\right);{\hat{S}}_{k_{j}})=1$         **else if** $0<|c_{i}|<l+2$ **then**             $p\left(O_{k}\left(x_{i}\right);{\hat{S}}_{k_{j}}\right)=\frac{|c_{i}|}{l+2}$         **end if**     **end if****end for**

**4. Relevance measure estimation:** The procedure is required to ensure timely tracking termination when an object leaves the field of view of the sensor system. It also serves to ignore false positive detections. The estimation corresponds to the data staleness counter $Q_{i}$, which reflects the number of consecutive measurements without a match. Its value is determined by Algorithm 2. The counter $Q_{i}$ is maintained individually for each sensor. If it exceeds a threshold in all sensors simultaneously, the object identifier is removed, and tracking is terminated in step 6. The relevance threshold $Q_{max}$ is set based on the dynamic characteristics of the observed objects and the frequency of sensor measurements. If the counter $Q_{i}$ equals zero in at least two sensors, the $Q_{i}$ values for all remaining sensors are also reset to zero.
**Algorithm 2** The pseudocode of the relevance measure estimation**Input:** $p(O_{k}\left(x_{i}\right);{\hat{S}}_{k_{j}})$ **Output:** $Q_{i}$**if **$p(O_{k}\left(x_{i}\right);{\hat{S}}_{k_{j}})=1$** then**     $Q_{i}\left(t\right)=0$**else if **$p(O_{k}\left(x_{i}\right);{\hat{S}}_{k_{j}})=0$** then**     $Q_{i}\left(t\right)=Q_{i}\left(t-1\right)+1$**else**     $Q_{i}\left(t\right)=Q_{i}\left(t-1\right)$**end if**

**5. Feature matching estimation:** Feature matching estimation is denoted as $p(O_{k}\left(x_{i}\right);{\hat{F}}_{k_{j}})$. It is calculated according to (2) if the feature is permanent (it does not change over time, for example, jersey colors or player’s jersey number).

(2) $$p\left(O_{k}\left(x_{i}\right);{\hat{F}}_{k_{j}}\right)=\left\{\begin{array}{cc} \frac{1}{l+2}, & \mathrm{if}\;m(F_{i};{\hat{F}}_{k_{j}})\approx 1\\ -\frac{1}{l+2}, & \mathrm{if}\;m(F_{i};{\hat{F}}_{k_{j}})\approx 0\\ 0, & \mathrm{if}\;F_{i}=0 \end{array}\right.$$

Feature matching estimation $p(O_{k}\left(x_{i}\right);{\hat{F}}_{k_{j}})$ is calculated as (3) if the feature is variable (which can change over time, for example, the color of the diode on the quadcopter). The type of feature is defined in advance.

(3) $$p\left(O_{k}\left(x_{i}\right);{\hat{F}}_{k_{j}}\right)=\left\{\begin{array}{cc} \frac{1}{l+2}, & \mathrm{if}\;m(F_{i};{\hat{F}}_{k_{j}})\approx 1\\ 0, & \mathrm{if}\;m\left(F_{i};{\hat{F}}_{k_{j}}\right)\approx 0\vee F_{i}=0 \end{array}\right.$$

The formulas use a similarity metric $m(F_{i};{\hat{F}}_{k_{j}})$, which quantifies the difference between the reference and the observed feature. The reference feature is initially assigned at initialization and may be updated at the sixth step of the algorithm, depending on its type.

**6. Matching between true and observed images:** Using the method of searching for the maximum element (4),

(4) $$O_{k}\left(x_{i}\right)=\max p\left(O_{k}\left(x_{i}\right);{\hat{O}}_{k}\right),$$

where $p(O_{k}\left(x_{i}\right);{\hat{O}}_{k}\left(x_{j}\right))$ is estimation of the match. It is convenient to represent it as a matrix, where the rows are $O_{k}\left(x_{i}\right)$, and the columns are ${\hat{O}}_{k}\left(x_{j}\right)$. The match estimation is calculated as the sum of two terms (5). The first term $p(O_{k}\left(x_{i}\right);{\hat{S}}_{k_{j}})$ is the match between $O_{k}\left(x_{i}\right)$ and ${\hat{S}}_{k_{j}}\in {\hat{O}}_{k}$. It was calculated in step 3 (Coordinate matching estimation). The second term $p(O_{k}\left(x_{i}\right);{\hat{F}}_{k_{j}})$ is the match between $O_{k}\left(x_{i}\right)$ and ${\hat{F}}_{k_{j}}\in {\hat{O}}_{k}$. It was computed in step 5 (Feature matching estimation).

(5) $$p\left(O_{k}\left(x_{i}\right);{\hat{O}}_{k}\left(x_{j}\right)\right)=p\left(O_{k}\left(x_{i}\right);{\hat{S}}_{k_{j}}\right)+p\left(O_{k}\left(x_{i}\right);{\hat{F}}_{k_{j}}\right)$$

In step 6, the true and observed images of objects are compared based on the search for the maximum correspondence score in the matrix. The maximum value is searched for in each of its rows, then the *i*-th true image is assigned the coordinates and features of the observed image of the corresponding *j*-th column.

In the case of multiple entries with the same maximum score in a given row, the match is assigned according to the tie-breaking rule $O_{k}\left(x_{i}\right)=S_{i}^{\prime }$, where $S_{i}^{\prime }$ denotes the projected predicted coordinates of the *i*-th object in the global coordinate system. Additionally, the data staleness counter is incremented at this iteration of calculations $Q_{i}\left(t\right)=Q_{i}\left(t-1\right)+1$. This situation can occur due to occlusion when objects are in close spatial proximity in the plane of the sensor field of view. But object tracks are constructed in the global coordinate system based on the true images (coordinates) of objects, obtained from multiple sensors, using multi-view geometry. Thus, a situation where two tracks in the global coordinate system correspond to a single observation in the plane of one sensor is allowed, as it reflects the real-world scenario.

If no observations are found for the true image of the previously tracked object, two situations are possible:

1. For some reason, the object $x_{i}$, located in the working area of one or more sensors, was not segmented at the measurement instant *t*.

2. The object $x_{i}$ left the working area of the sensor system.

These situations are resolved as follows. If the counter $Q_{i}$ exceeds the predefined threshold for all sensors simultaneously, the track for the object is terminated due to prolonged absence of observations. The object’s identifier is removed, and the total number of tracked objects is decremented. Otherwise, the coordinates of the true image of the *i*-th object will be equal to the projected predicted coordinates from the global coordinate system with the corresponding identifier, and it will also be assigned a reference feature.

Coordinate reconstruction (3D reconstruction) and coordinate projection are performed using known methods. An example of the calculation is provided in [20].

Given that the algorithm is designed for tracking within a global coordinate system, the initialization or search for new objects occurs over several iterations of calculations. It is necessary because a new object may initially appear and move within the measurement range of only one sensor operating in a single plane, making coordinate reconstruction in a global coordinate system impossible. Additionally, there is a risk of mistakenly identifying a false positive segmentation as a new object. To avoid this error, but at the same time initialize a new object, the following steps are implemented:

1. If the coordinates-matching estimation identifies potential new objects, they are assigned new identifiers. Subsequently, a coordinate reconstruction is performed.

2. The obtained coordinates are then projected back into the sensor’s coordinate system.

3. During the clustering step, if the measured coordinates align with the back-projected coordinates, it is concluded that a new object has been detected. One of the previously assigned identifiers is allocated to this object, while any redundant identifiers are discarded.

As a special case, initialization may be performed before the main tracking algorithm starts. It is particularly useful in scenarios such as sports events, where the number of objects is known to remain constant. In this case, position observations from all sensors are fused for 3D reconstruction. Reference features are predefined, and all the elements $Q_{i}$ are initialized to zero.

## 5. Experimental Results

The first experiment utilizes the proposed algorithm in its original form to track players during a basketball game, enabling a direct comparison with state-of-the-art tracking methods on the open-source APIDIS dataset. The video sequences are acquired from a stationary multi-camera system.

In the second experiment, the tracking of airborne objects (ultralight aircraft and birds) is employed to address thermal updraft localization, as soaring targets utilize the same thermals to maintain and gain altitude. In this context, a modified form of the algorithm is employed, tailored to demonstrate operation with a single moving wide-angle camera in a specific scenario. This is an example of how a single moving camera can emulate the operation of a pair of sensors.

### 5.1. Tracking Multiple Objects by the Multi-Camera System

**Figure 3:**
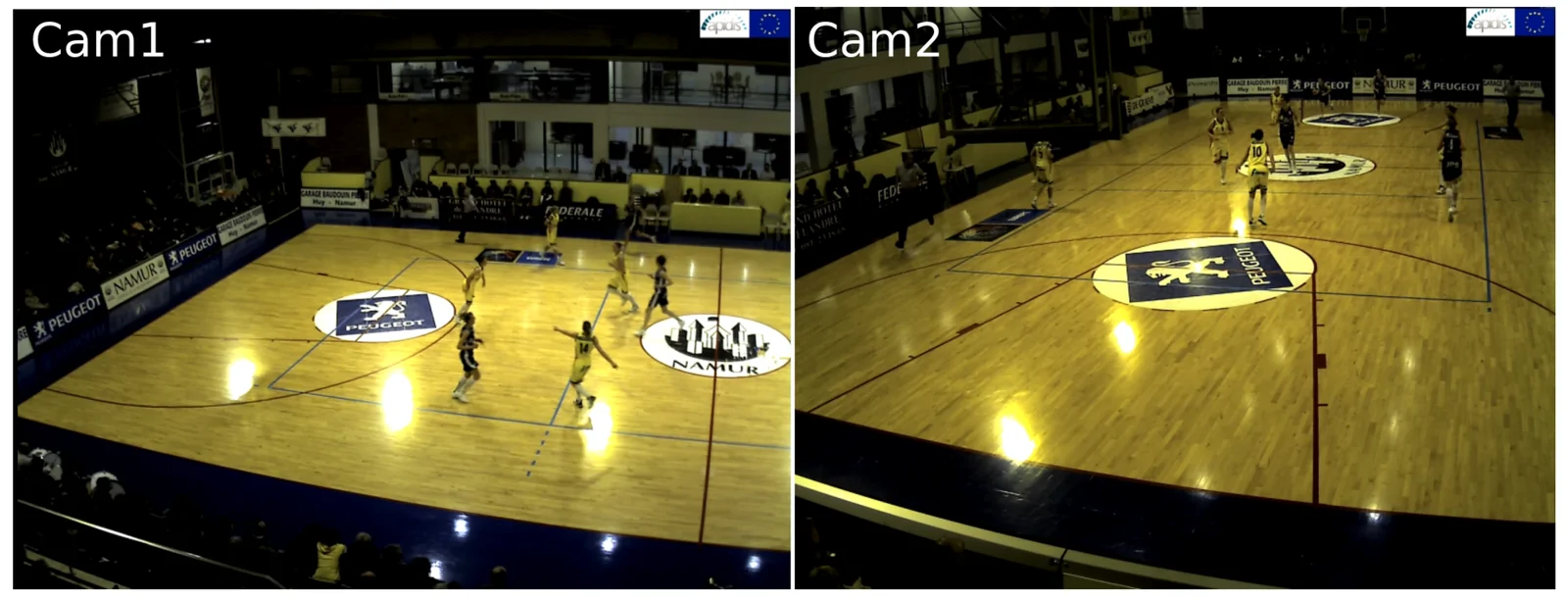
Frames from camera 1 (Cam1) and camera 2 (Cam2).

The publicly available APIDIS dataset was used for direct comparison of the developed algorithm with known analogs. This dataset includes a video recording of a basketball game captured by a system of cameras positioned around the playing court. Both intrinsic and extrinsic camera calibration parameters are provided. Examples of frames obtained from cameras 1 and 2 are presented in Figure 3.

The experiment involved cameras 1, 2, 3, and 6, which monitored 12 objects (5 players in two teams and 2 referees). The detection task was challenging due to the intense dynamics of the scene, bad lighting conditions, and a complex background. Segmented data were obtained using the source [21]. Jersey color data were used as permanent additional features. The experiment used the Mean Shift clustering method with a bandwidth of 15, $Q_{max}=10$. The chosen Kalman filter model is presented in [18], the variance of acceleration noise $\sigma^{2}=100$, the noise level of measurement results from the sensor used r=4.

Tracking was performed over 60 s. During this time, 1500 frames were processed, which means the cameras were pseudo-synchronized at a frame rate of 25 fps. Within this timeframe, all objects moved from the right side of the court to the left and then returned to their original zone.

**Figure 4:**
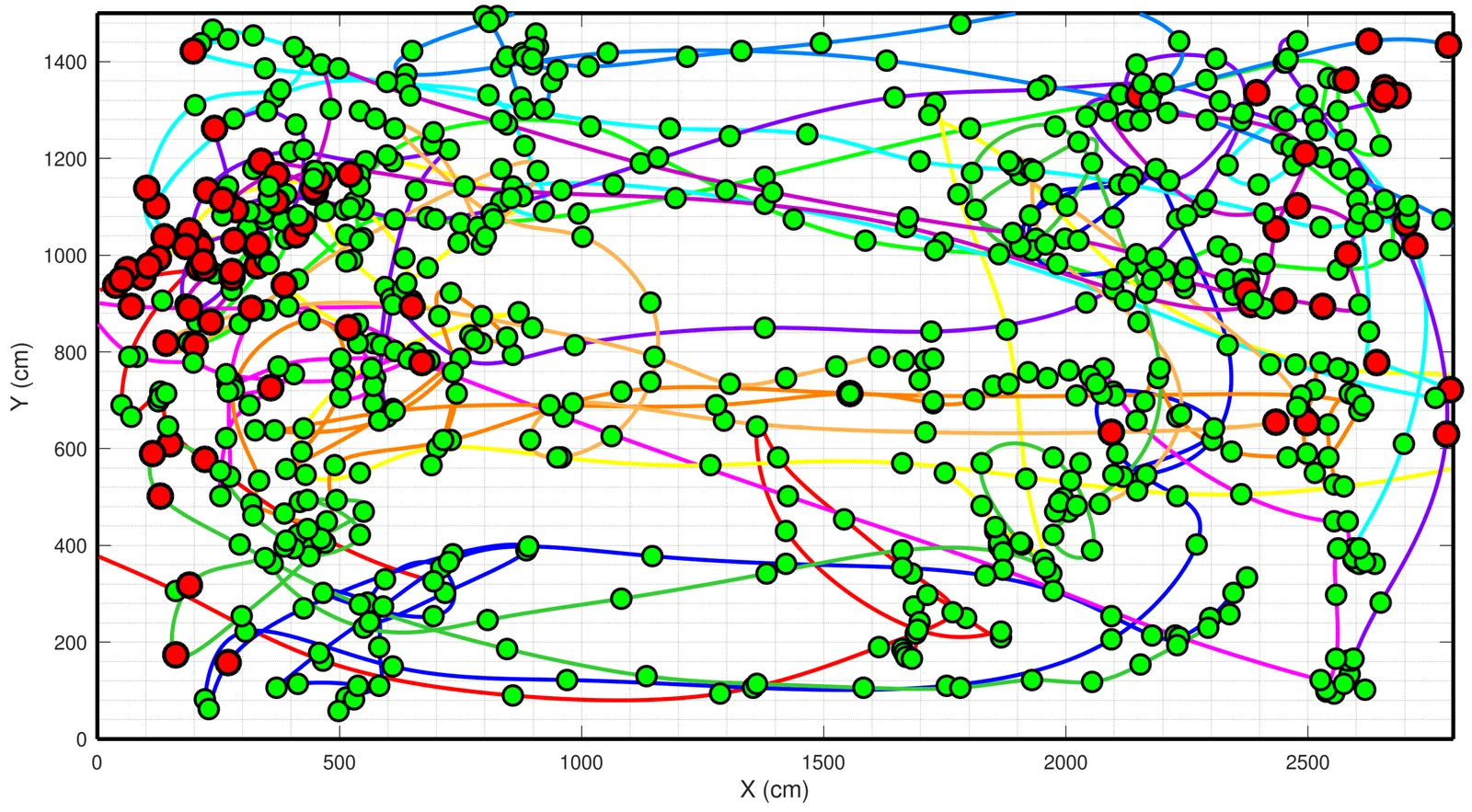
Tracking results.

The dataset provided ground truth positions of the players on the field, recorded at one-second intervals (60 positions in total for each object). The results of the tracking algorithm are illustrated in Figure 4. Green markers indicate successfully matched object–ground truth pairs, while red markers represent ground truth positions that were not matched in the tracking results. For enhanced trajectory visualization, the ground truth positions shown in Figure 4 have been interpolated. Each object is represented by a distinct color to facilitate differentiation.

In this experiment the multiple object tracking accuracy (MOTA) metric was used for comparison, which is calculated as (6), where ${\mathrm{FP}}_{t}$—the number of false positives at measurement instant *t* (objects detected by the model but absent from the ground-truth data), ${\mathrm{FN}}_{t}$—the number of false negatives (objects present in the ground-truth data but not detected by the model), ${\mathrm{IDSW}}_{t}$—the number of identity switches (instances where a track is incorrectly switched from one object to another), $G_{t}$—the total number of ground-truth (true) objects at measurement instant *t*.

(6) $$\mathrm{MOTA}=1-\frac{\sum_{t}\left({\mathrm{FP}}_{t}+{\mathrm{FN}}_{t}+{\mathrm{IDSW}}_{t}\right)}{\sum_{t}\left|G_{t}\right|}$$

Table 2 presents the results of a comparison using the MOTA metric with current methods for tracking moving objects, employing the APIDIS dataset. Based on these results, the proposed solution demonstrates high performance and is suitable for use in tracking systems.

In work [23], the MOTA score of 85.8% was obtained. However, in that case, the tracking targets were strictly predefined, and cameras aimed at one half of the playing field were used for tracking. The main goal of the experiment was primarily to demonstrate the feasibility of using cameras with different focal lengths. In the present work, major changes to the algorithm were made. In the experiment, tracking was performed for all objects moving across the entire playing field.

### 5.2. Thermal Updrafts Localization

For this experiment, a custom dataset was used. It includes video recordings captured at different times with a GoPro MAX camera (GoPro, Inc., San Mateo, CA, USA) by a professional paraglider pilot during a multi-hour flight. The duration of each video ranged from 5 to 8 min, with a frame rate of 60 fps. The intrinsic camera parameters were computed in advance. The extrinsic parameters, including position and orientation angles, were derived from GPS and IMU data.

The distinctive characteristic of this scenario is the spatial separation between the soaring objects. To prevent collisions, the targets maintain different distances in both the horizontal plane and altitude. For this scenario, a special case of the proposed algorithm was utilized. The Kalman filter, which introduces additional coordinates, was not applied. The approach is justified in terms of the efficiency and performance of the algorithm. The computations were performed using the values obtained over the entire time period. For clustering, a hybrid Mean Shift and Grid-DBSCAN approach with a grid resolution of 200 cells was employed.

Segmented data were obtained using the YOLOv8 neural network. It shows the best performance in detecting small aerial objects in the image due to the long shooting distance [24]. The model was pre-trained on publicly available datasets containing images of birds and paragliders. During further analysis, the type of a specific object was used as an additional feature.

**Figure 5:**
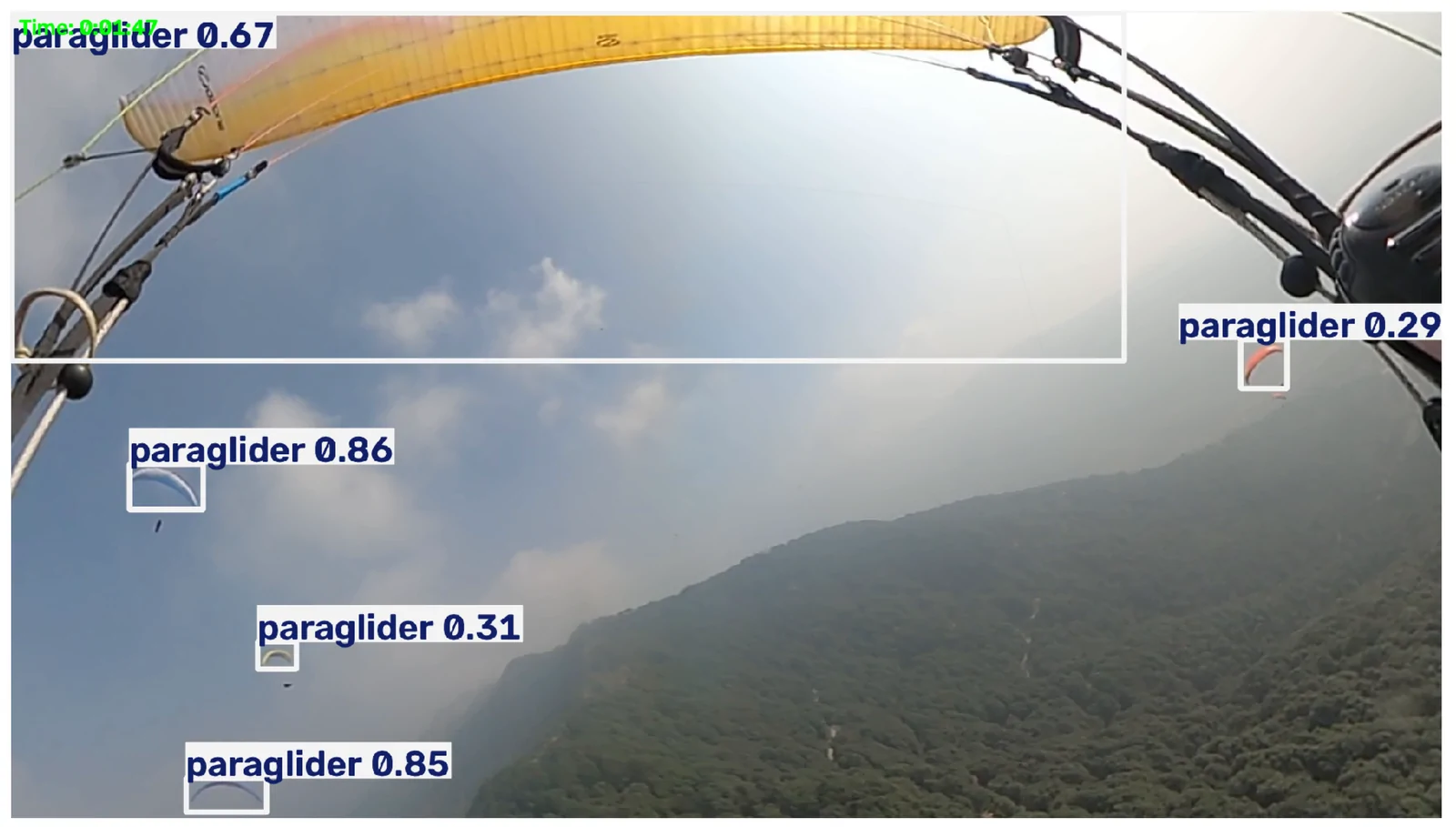
Example of paraglider segmentation using YOLOv8.

A screenshot from the custom dataset is shown in Figure 5. It clearly demonstrates that the operator’s paraglider wing entered the frame and was detected, which could lead to an erroneous result. However, thanks to step 4 of the proposed algorithm, duplicate data is removed.

After detection, the algorithm projects the objects onto a common plane and then clusters them. The objects are grouped based on the proximity of their coordinates and the similarity of their motion trajectories. Clusters are considered potential indicators of thermal updrafts.

Note separately that the experiment assumes the feasibility of simulating stereovision capabilities for distance estimation to remote objects using a single mobile camera. This methodology is justified by the specific requirements and environmental constraints of the task: the required localization accuracy is moderate, and target objects are situated at considerable ranges. For the task, the camera used features high resolution and frame rate.

**Figure 6:**
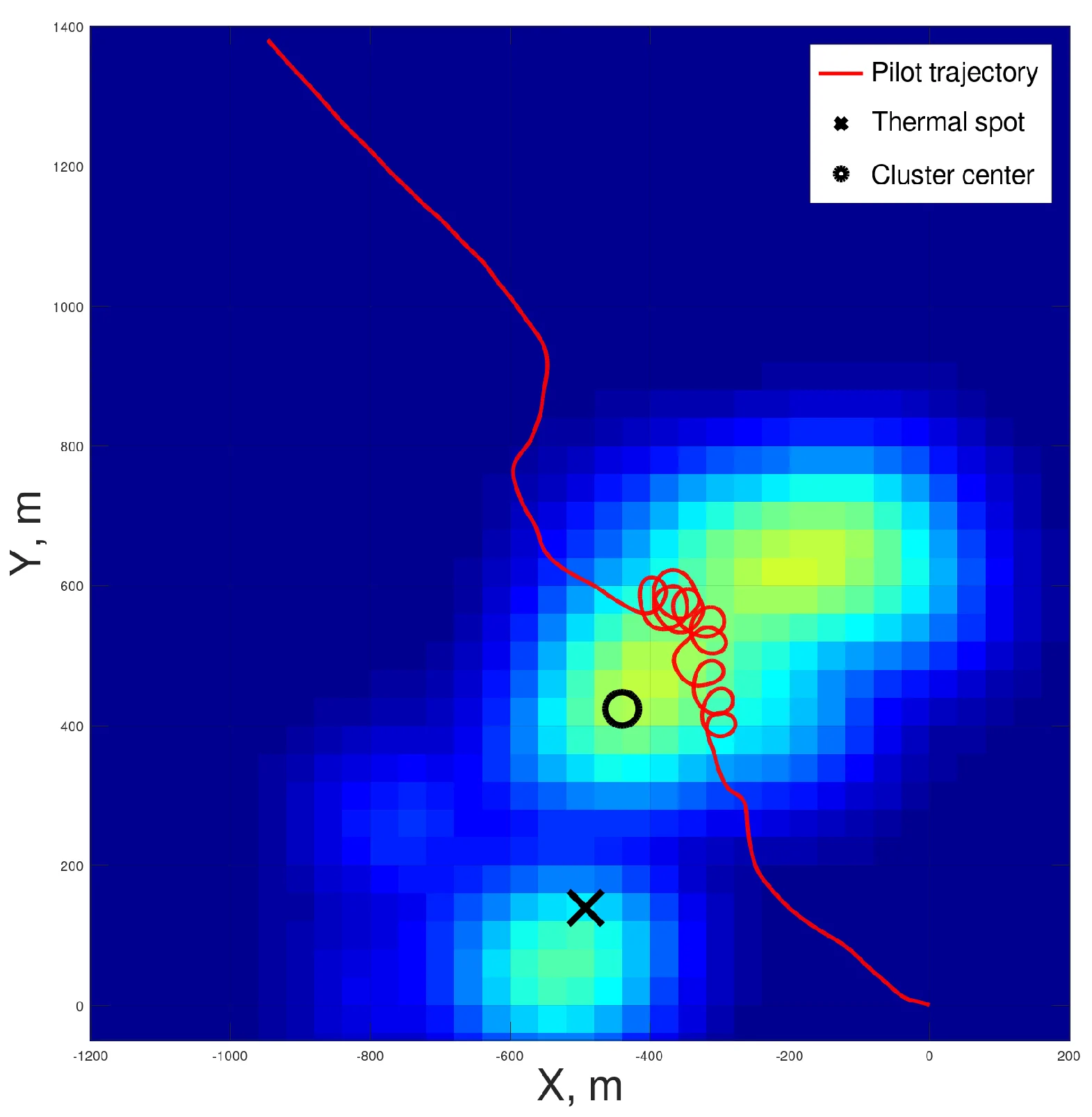
Thermal updraft localization results based on a video sequence of 5 min.

**Figure 7:**
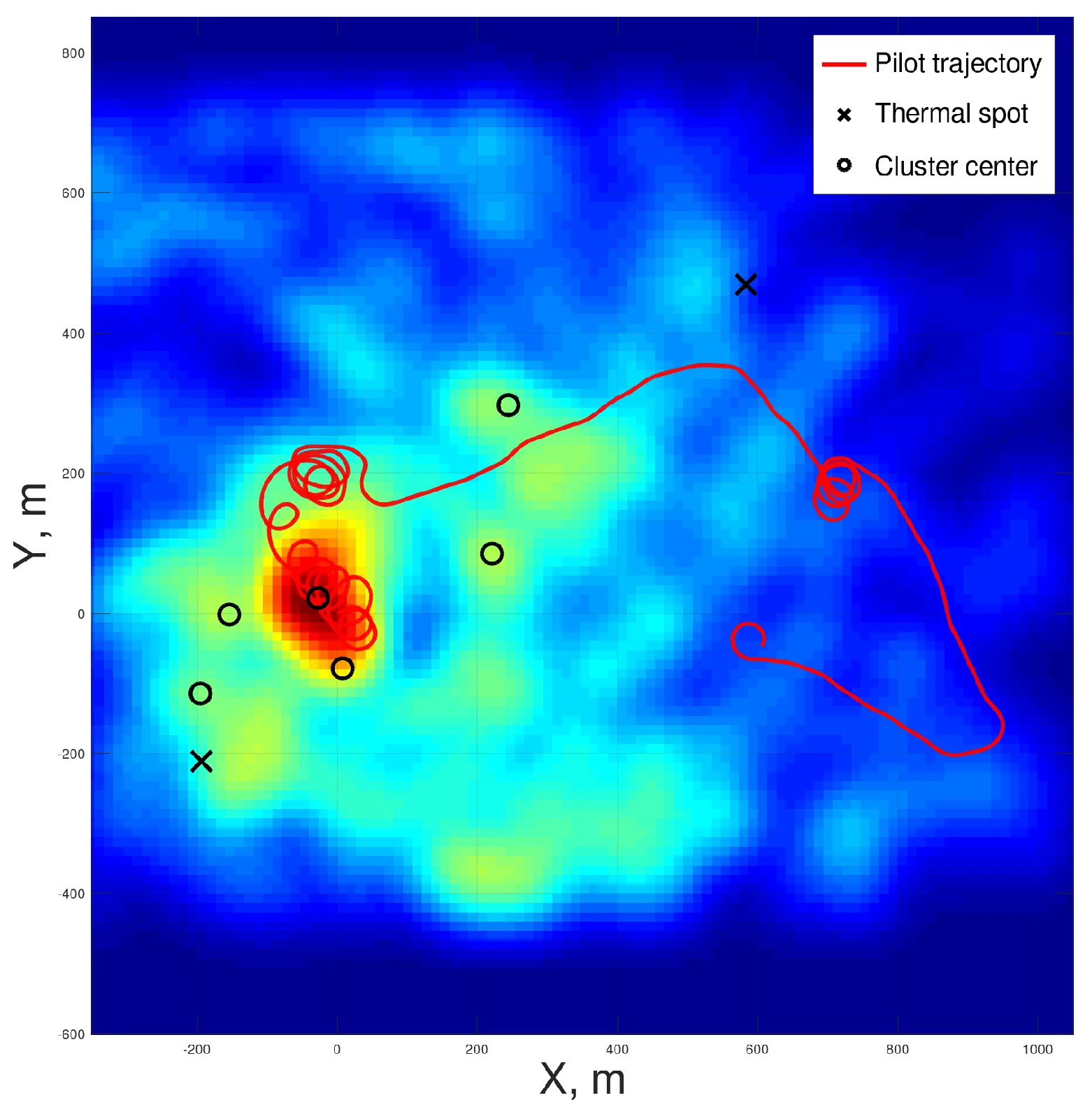
Thermal updraft localization results based on a video sequence of 8 min (with a small number of surrounding objects).

**Figure 8:**
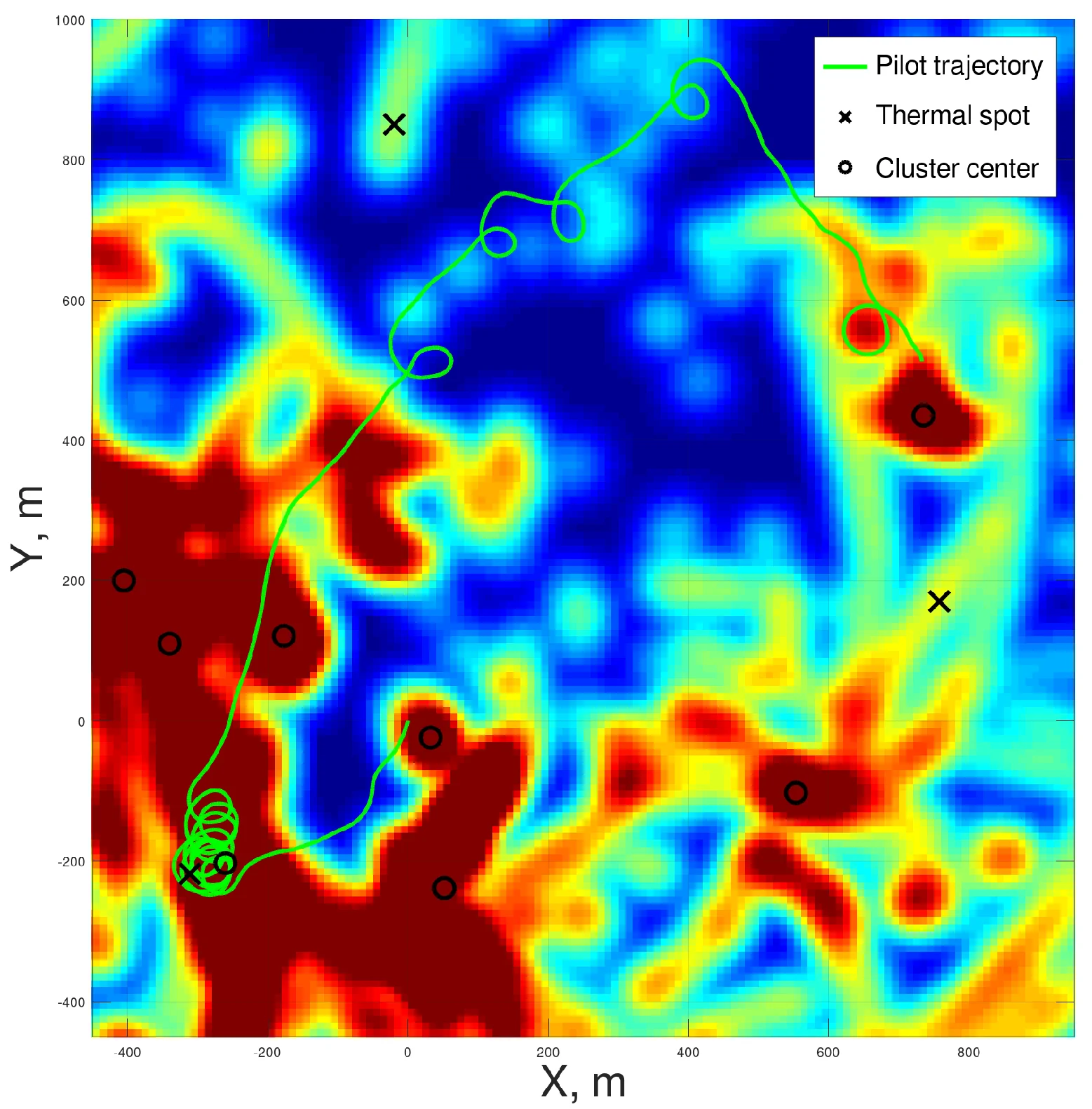
Thermal updraft localization results based on a video sequence of 8 min (with a large number of surrounding objects).

The heatmap in Figure 6, Figure 7 and Figure 8 illustrates the concentration of objects in space. The visible area was divided into cells of 10 × 10 m. Then, the maximum density value was determined, and the values for all other cells were normalized relative to it. In addition it presented: the pilot’s trajectory (the GPS telemetry recorded in the video was converted to meters on the plane, with the origin set at the starting point), thermal spot (current data on the probabilistic distribution of thermal updrafts based on historical observations and averaged models are available through a specialized cartographic service; the coordinates were projected onto the same local plane similarly) and cluster center.

The formation and dynamics of thermal updrafts depend on the heterogeneity of the underlying surface, instantaneous changes in weather conditions, turbulence, and time of day, leading to significant discrepancies between the probabilistic (from the cartographic service) and actual locations of thermals. A paraglider pilot can visually identify an updraft by the characteristic circling of soaring objects in the air. Therefore, it seems promising to use visual feedback during the automatic control of an unmanned aerial vehicle for the search and localization of thermal flows. The results of the experimental studies conducted in this work confirm the following assumption: when the largest concentration of objects is localized, it is advisable to search for the thermal flow in that very area. Moreover, if there are several points with objects, an experienced pilot will head towards the area with the greatest concentration of such objects.

## 6. Discussion

The paper proposes a multiple object tracking solution applied at the data association stage to mitigate errors from the segmentation and detection stages.

Often, the main challenges are considered only in the context of processing segmented video data. However, their composition does not change even if other types of sensors, such as rangefinders, are used. This generality creates prerequisites for the development of unified tracking algorithms that could be used to solve a wide range of tasks, regardless of the actual composition of the sensor system. At the same time, combining video data with various sensors that provide depth information opens up additional opportunities to improve tracking quality, primarily by addressing occlusions. Therefore, to begin with, a generalized task statement for tracking moving objects via a multisensor system is formalized, abstracting away from specific sensor and object types and counts.

The novel tracking algorithm using segmented data analysis is presented to solve the stated generalized task. The operations of coordinate reconstruction, conversion to common units of measurement, coordinate projection, as well as the prediction and correction steps of Kalman filters can be performed independently for each observed object (Figure 2). Such an architecture opens up broad prospects for hardware acceleration through massive computational parallelism. Therefore, the most promising solution for the physical implementation of the proposed algorithm is a distributed system. Since the segmentation procedure is treated as a separate step, it can also be executed independently. Therefore, sensors equipped with an integrated processor can be utilized for primary data processing, segmentation, and, in some cases, object recognition tasks. Such an approach significantly accelerates data processing. This is highly relevant for the development of the tracking system.

The proposed algorithm was evaluated on diverse datasets acquired from a stationary multi-camera system and a single mobile camera. In the first experiment, the MOTA score of 81.8 % was obtained on the public APIDIS dataset. As illustrated in Figure 4, the highest concentration of red (error) markers occurs at the court edges. This is attributed to the camera placement, the multi-view geometry approach employed in the algorithm, and the single segmentation method provided with the dataset. The algorithm requires segmented data from at least two sensors for correct operation. At the same time, data can be obtained using various segmentation methods for each sensor, depending on environmental conditions. In the case of a prolonged absence of these data, the coordinate prediction accuracy decreases for objects that abruptly change direction.

In the second series of experiments, thermal updrafts were localized. Notably, the review [25] points out that visual feedback has not been applied in thermal seeking perception techniques. This work confirms the applicability of the proposed algorithm for determining the flight direction of an autonomous glider to gain altitude. The results show that a professional paraglider pilot primarily relies on the actual concentration of soaring objects. To a lesser extent, the pilot relies on historical statistical data about the location of thermals, available through specialized services.

Thus, the advantages of the proposed algorithm are as follows:

1. Flexibility across different tasks. The algorithm is designed for a generalized task. Its structure allows it to operate with diverse data samples and provides flexibility in hardware implementation.

2. The number of targets is not fixed in advance and can change during the tracking process. Timely tracking termination is ensured for targets that have left the working zone of the sensor system.

3. Segmentation can be performed by both modern methods (based on YOLOv8, 2023) and traditional approaches (based on a legacy technique, 2009). In the latter case, a competitive tracking performance is still achieved.

Future work will address two key directions. First, the impact of the sensor system composition and its spatial configuration on tracking accuracy will be evaluated within the framework of the proposed algorithm. Second, the approach will be extended by incorporating the analysis of vertical velocity and the assessment of object climb rates. This will enhance the applicability of the solution for autonomous glider operations. The algorithm will be used not only for localizing thermal updrafts to ensure altitude gain, but also for supporting the glider’s motion directly within the updraft, where the risk of collision with soaring objects exists.

## Figures and Tables

**Table 1:** List of notations.

Notation	Description
*K*	Number of sensors
*N*	Number of moving objects
*J*	Number of observations
*U*	Number of clusters
$x_{i}$	State vector of the *i*-th object
$x_{i}^{\prime }$	Predicted state vector of the *i*-th object
*i*	Object index
*k*	Sensor index
*j*	Observation index
*n*	Cluster index
*t*	Measurement instant
*S*	The spatial object position
*V*	Velocity vector
*a*	Acceleration vector
$F_{i}$	Additional feature vector of the *i*-th object
${\hat{S}}_{\mathrm{kj}}$	Centroid coordinates of the *j*-th detected object from the *k*-th sensor
$S^{\prime }$	Projected predicted coordinates
$\bar{S}$	Predicted object coordinates
${\hat{F}}_{\mathrm{kj}}$	Numerical attribute of the additional feature for the *j*-th observation from the *k*-th sensor
*A*	State transition matrix
${\hat{O}}_{k}\left(x_{i}\right)$	Observed image of the *i*-th object obtained from the *k*-th sensor
$O_{k}\left(x_{i}\right)$	True image of the *i*-th object at the *k*-th sensor
*l*	Number of sensors from which predicted coordinates are projected
$C_{n}$	The *n*-th cluster obtained by applying a clustering method
$c_{i}$	Set of coordinates associated with object index *i*
$Q_{i}$	Data staleness counter
$Q_{max}$	Relevance threshold

**Table 2:** Comparison of tracking algorithms using the MOTA metric.

Tracking Algorithm	MOTA
Online Multiple Athlete Tracking with Pose-Based Long-Term Temporal Dependencies [16]	75.2
Online Multiplayer Tracking by Extracting Temporal Contexts with Transformer [22]	75.3
Variational inference for 3-D localization and tracking of multiple targets using multiple cameras [13]	79.6
Multi-camera multi-player tracking with deep player identification in sports video [17]	81.1
Developed algorithm	81.8

## Data Availability

The data that support the findings of this study are available from the corresponding author upon reasonable request.

## Abbreviations

The following abbreviations are used in this manuscript:

FPS	Frames Per Second
GPS	Global Positioning System
IMU	Inertial Measurement Unit
JPDA	Joint Probabilistic Data Association
LMB	Labeled Multi-Bernoulli
MAP-EM	Maximum A Posteriori Expectation-Maximization algorithm
MHT	Multiple Hypothesis Tracking
MOTA	Multiple Object Tracking Accuracy
PHD	Probability Hypothesis Density
YOLO	You Only Look Once
